# Association of *TSHR* gene single nucleotide intronic polymorphism with the risk of hypothyroid and hyperthyroid disorders in Yazd province

**DOI:** 10.1038/s41598-022-19822-0

**Published:** 2022-09-21

**Authors:** Fahime Sadat Naghibi, Seyed Mohsen Miresmaeili, Amaneh Javid

**Affiliations:** Department of Biological Sciences, Faculty of Science and Engineering, Science and Arts University, Yazd, Iran

**Keywords:** Biochemistry, Biological techniques, Chemical biology, Genetics, Immunology, Molecular biology

## Abstract

The present study was carried out, for the first time, to evaluate the association of *rs2268458* polymorphism, biochemical and environmental factors on hypothyroid and hyperthyroid disorders in thyroid patients and healthy individuals in Yazd province, Iran. In this study, blood samples were collected from a total of 100 cases, including 60 hypothyroid, 20 hyperthyroid and 20 normal individuals. DNA was extracted from blood samples and the *rs2268458* single nucleotide intronic polymorphism was evaluated using Restriction Fragment Length Polymorphism PCR (RFLP-PCR). The results have shown that 59 individuals were homozygote (TT), 40 cases were heterozygote (TC) and one homozygote (CC) case. Of 59 TT homozygote cases, 25 cases were hypothyroid females and 7 hypothyroid male patients. While, heterozygote TC group consisted of 20 hypothyroid females and 7 hypothyroid male cases. Furthermore, only 1 (CC) homozygote male hypothyroid patient was observed in this study. The hyperthyroid population consisted of 7 (TT) homozygote hyperthyroid female cases, 8 (TC) heterozygote hyperthyroid female cases, 3 (TT) homozygote hyperthyroid male cases and 2 (TC) heterozygote hyperthyroid male cases. According to our study, heterozygote cases (TC) showed less severe symptoms, while homozygote cases (TT) showed no serious symptoms and the (CC) homozygote case showed severe thyroid abnormalities. So, it can be concluded that the TSHR-related rs2268458 polymorphism is associated with hypothyroidism and hyperthyroidism in the male and female populations of Yazd Province, Iran and C allele can be a risk factor for some physio-biochemical and hormonal imbalance in the thyroid disorder patients.

The thyroid gland influences almost all of the metabolic processes in human body. Thyroid-related disorders can vary widely from a small, harmless goiter (enlarged thyroid gland) that needs no specific treatment to life-threatening cancer which might need invasive treatment. Thyroid diseases rank as the most prevalent endocrine disorders after diabetes^1^. The thyroid gland secretes hormones that help regulate the body’s metabolism. According to a recent study, thyroid-related diseases affect 200 million people worldwide, of which about 60% are unaware of their disease. There are several factors involved in thyroid dysfunction, the most common of which is iodine deficiency that is estimated to be affecting 2 billion individuals worldwide and is thought to cause primary hypothyroidism^2^. The most common cause of hypothyroidism is chronic autoimmune thyroiditis, also known as Hashimoto’s disease (HT). High concentrations of anti-thyroid antibodies (predominantly thyroid peroxidase (TPO) antibodies and anti-thyroglobulin antibodies) are present in most patients with autoimmune thyroiditis^3^. Histological and cytological features of HT constitute a dense thyroidal accumulation of lymphocytes, plasma cells and occasional multinuclear giant cells. HT can be seen with various severities from a life-time hypothyroid state to a euthyroid. It was demonstrated that even having a parent with Hashimoto’s disorder can put the child at the risk of thyroid disorders, such as; thyroid carcinoma as it has been examined on a 15-year-old girl suffering from hyper-functioning papillary thyroid carcinoma^4^.

Thyroid hormones play an imperative role in human physiology, metabolism, growth, development and etc. Therefore, they are taken into consideration when it comes to endocrine disorders. Thyroid disorders are regarded as a group of pre-dominantly biochemical dysfunctions because they sometimes lack specific symptoms to be defined^5^. Overt or clinical primary hypothyroidism is defined as the disorder which has above-the-range concentrations of thyroid-stimulating hormone (TSH) and below-reference concentrations of free-thyroxine. While, mild or subclinical hypothyroidism has both above-the-range TSH and normal free-thyroxine levels; and it is considered as a sign of early thyroid failure. In patients with subclinical hypothyroidism, TPO antibodies' testing can be a beneficial option to predict the progression to overt hypothyroidism^6^.

Thyrotropin-releasing hormone (TRH) is secreted by hypothalamus gland of central nervous system (CNS) and promotes the secretion of thyroid stimulating hormone (TSH) by pituitary gland^7^. TSH regulates thyroid function by stimulating the secretion of triiodothyrine (T3) and thyroxine (T4) hormones by the thyroid gland. There is inadequate knowledge about the association of genetic variations and thyroid hormone metabolism in human population, which has led a number of questions unanswered about thyroid dysfunctions and how to handle them^8^. The TSH receptor (TSHR) is a 7-transmembrane domain G protein–coupled receptor (GPCR) that is a major thyroid-gland regulator and is a key auto-antigen in autoimmune thyroid diseases (AITDs)^9^. The *TSHR* gene is located on chromosome 14q and is considered to be related to disease-specific functions. Genetic variants in the TSHR gene were recognized to be associated with the risk of thyroid diseases^10–12^. It is unclear that whether TSHR function and thyroid disorders, such as; Graves' disease (GD) are relatively associated or not, and that TSH screening has facilitated their diagnosis.

The immunologic processes in GD are relatively relevant to TSHR and this disease exhibits autoantibodies with both linear and conformational epitopes. There are three types of TSHR antibodies: stimulating, blocking and cleavage antibodies, and each type exhibits different functional capabilities in Graves’s disease (GD). Anti-TSHR agonistic antibodies have a pathogenic role in GD as they cause lack of TSHR tolerance and contribute to GD pathogenesis^13^. Besides, numerous studies have indicated that mutations in TSHR and Gαs genes are associated with thyroid disorders^14^. Previous studies have shown that chromosome 14 is associated with thyroid disorders; particularly hypothyroidism and its most important gene-regions are 35 Mbp, 95 Mbp and 93 Mbp, in close proximity to the *rs2268458* SNP^15–18^, which is located on intron 1 of TSHR gene.

Keeping in mind the significance of TSHR and its altered function in thyroid metabolism and pathology, the present study was focused on investigation of the association of SNP rs2268458 at the TSHR gene locus with hypothyroidism and hyperthyroidism in male and female populations of Yazd Province, Iran. Furthermore, we analyzed the association of genotypes, different age and sex groups with AITDs.

## Materials and methods

### Study population

A population-based cohort study was performed during the years 2019–2020 in Yazd, a metropolitan city of Central Iran. In short, male and female (*n* = 80) hypothyroid and hyperthyroid patients, and 20 healthy candidates (in total = 100 candidates) were selected and acquired the informed consent to participate in the study. The present study was approved by Human Research Ethics Committee; Academic Center for Education, Culture and Research (ACECR), Iran and the research was conducted in accordance with the Declaration of Helsinki. These patients were examined by a trained general practitioner and an endocrinologist. All data including demography, age, past history of thyroid and any other disorders, smoking history and use of medications were collected. The patients were also examined for goiter or nodules. The body mass index (BMI) was calculated by dividing weight (kg) to the height square (m^2^).

### Blood sample collection and genomic DNA extraction

Fasting blood samples were collected from hypo- and hyper-thyroid patients who matched the study criteria. Blood (5 mL) was withdrawn and transferred into plain tubes (3 mL) and EDTA tubes (2 mL). The blood samples in the plain tube were centrifuged after 30 min of sampling for serum collection, which was then stored at − 20 °C and stored for further analysis. Samples were assayed in duplicate and the mean of the paired results were analyzed. EDTA tubes were stored at − 20 °C for genomic DNA extraction. Genomic DNA was extracted and purified from whole peripheral blood samples using Blood Genomic DNA Extraction Kit (Pars Tous Biotechnology Co; Mashhad, Iran). DNA sample were stored at − 86 °C in aliquots for further analysis.

### Biochemical analysis of blood samples

Blood samples (5–10 mL blood, mixed with EDTA (each mL of blood was mixed with 60 μL of EDTA)) were taken and T3, T4, and TSH measurements were carried out. Serum total T4 and T3 were analyzed by radioimmunoassay. Normal range of T4 concentration was 4.5–12.0 μg/dL and T3 normal concentration was 0.92–2.79 nmol/L. Serum TSH concentration was assessed by immunoradiometric assay. The normal range for TSH was 0.3–3.6 mIU/L. The hypothyroid individuals were described as follows: overt (clinical) hypothyroidism (TSH > 10 mIU/L), subclinical hypothyroidism (10 ≥ TSH > 3.6 mIU/L). While, hyperthyroidism was described as overt (or clinical) hyperthyroidism (TSH level < 0.1 mIU/L and total T4 > 12 μg/dL and/or total T3 > 2.79 nmol/L) and subclinical hyperthyroidism (TSH < 0.3 mIU/L and total T4 and total T3 within normal range, 4.5–12.0 μg/dL and 0.92–2.79 nmol/L, respectively).

### TSHR-SNP-rs2268458 analysis by PCR–RFLP

PCR amplification of the TSHR gene was carried out using PCR Master Mix (Taq DNA Polymerase Master Mix, Ampliqon A/S, Odense, Denmark). Based on the analysis of^19^, we examined *TSHR*-SNP-rs2268458, located in intron 1, using standard RFLP-PCR protocol^19^. Human genomic DNA (about 25 ng) was amplified by PCR machine and 415-bp products were generated, as described above. The primer sequences used for this analysis are as follows:

$$\begin{aligned} & {\text{F}}{:}\;{5}^{\prime}\;{\text{CTAACCAGCAGAGGGAGCAC3}}^{\prime} \\ & {\text{R}}{:}\;{5}^{\prime}\;{\text{CCACTGCTTAAAGCCCAGAT3}}^{\prime} \\ \end{aligned}$$

These primers flanked the DNA segment in *TSHR* gene intron 1. PCR sample consisted of 0.2 mg genomic DNA, 0.5 mL Taq polymerase, 0.5 μM primary forward, 0.5 μM primary reverse and 200 mM dNTP. The DNA samples were amplified for 35 cycles with initial denaturation at 94 °C for 5 min, annealing cycle at 53 °C for 30 s, extension cycle at 72 °C for 30 s, denaturation at 94 °C for 30 s and final extension was carried out at 72 °C for 7 min^20,21^. Concentration and DNA purity was calculated using nanodrop with a purity range of 1.8–2.0 (ScanDrop^2^, Analytik Jena, Germany). The final amplification mixtures were then electrophoresed on 2% agarose gel for 45 min at 90 V. Visualization of the DNA fragment bands was done using Gel documentation system. Then, 8 μL of PCR products was digested for 2 h in 10 μL total volume with the restriction endonuclease Alu I (Therno Fisher Scientific Inc.) overnight according to the manufacturer's instructions. RFLP-PCR was carried out to varify the wild allele and the *rs2268458* SNP variant with Alu I restriction enzyme along with a undigested DNA control. After digestion, the digested fragments were loaded onto 2% agarose gel and visualized by ethidium bromide and gel documentation to carry out genotype pattern analysis. Since Alu I digestion determines AGTT versus AGCT, this allowed the determination of each individual's hetero- or homo-genotype.

### Exclusion criteria

The patients not fulfilling our study criteria and AITD subjects having other underlying diseases, thyroid cancer, renal failure, mental illness, liver failure, systemic diseases or chronic respiratory diseases were excluded from this study.

### Statistical analysis

The resultant data were analyzed using SPSS (version 22.0). In bivariate analysis, the numeric variables were calculated using *t*-test when there was a normal distribution and Mann Whitney/Kruskal Wallis test for abnormally distributed data. The bivariate analysis for categorical variables used χ^2^-squared test. For the multivariate determination, the logistic regression was used as the dependent variables were also categorical variables.

## Results

**Figure 1:**
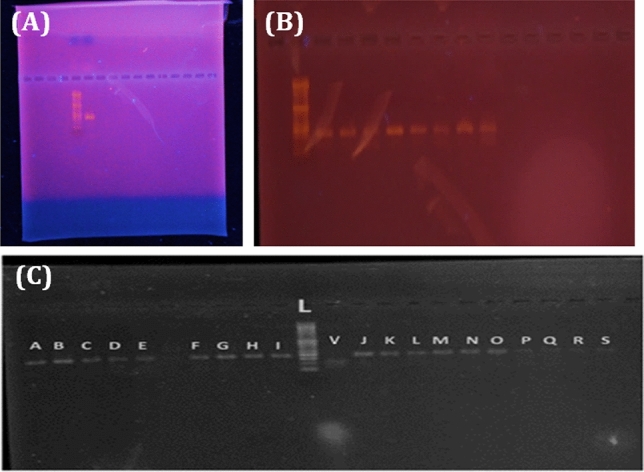
(**A**) The pattern of electrophoresis gel of extracted DNAs. (**B**) The pattern of PCR product of TSHR gene fragment and (**C**) the pattern of PCR products on agarose gel electrophoresis, related to TSHR gene (162 bp band) after being restricted by Alu I enzyme. The normal cases (B, C, N, O and P), the hyperthyroid cases (F and R) and the hypothyroid cases (A, D, E, G, H, I, V, J, K, L, M and S). Heterozygote genotypes (TC) with 162,100 and 62 bp bands: (D, E, F, G, I, J, L, N, M and S). Homozygote genotypes (TT) with a 162 bp band (A, B, C, H, K, O, P, Q and R). Homozygote genotype (CC) with 100 and 62 bp bands (V).

The extracted DNA was clear and not fragile (Fig. 1A). PCR product of TSHR locus showed a single 162 bp band (Fig. 1B). Figure 1C shows the pattern of PCR products on agarose gel electrophoresis, related to TSHR gene (162 bp band) after being restricted by Alu I enzyme. The normal (bands B, C, N, O and P), the hyperthyroid (bands F and R) and the hypothyroid cases (bands A, D, E, G, H, I, V, J, K, L, M and S) were analyzed in this experiment. Heterozygote genotypes (TC) were shown by 162, 100 and 62 bp bands in D, E, F, G, I, J, L, N, M and S samples. Homozygote genotypes (TT) were characterized by a 162 bp band in A, B, C, H, K, O, P, Q and R samples. Homozygote genotype (CC) was shown with 100 and 62 bp bands in V sample.

**Figure 2:**
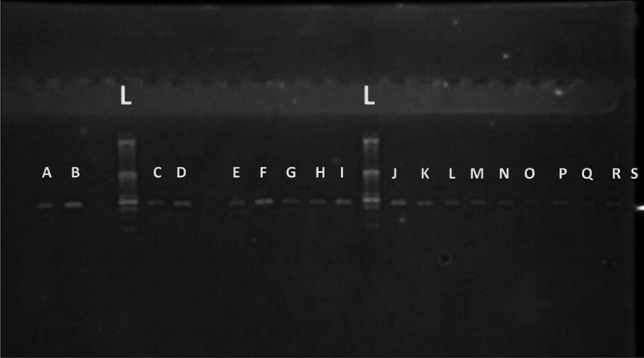
The pattern of PCR products on agarose gel electrophoresis, related to TSHR gene (162 bp band) after being restricted by Alu I enzyme. The Q, O, N, K, J, I, G, E, C and B cases with heterozygote genotype (TC) have 162, 100 and 62 bp bands. The R, P, M, L, F, D and A cases with homozygote genotype (TT) have 162 bp band.

According to Fig. 2 the PCR products were treated by Alu1 enzyme. The B, C, N, O, P cases were normal, the R and F cases were hyperthyroid and the A, D, E, G, H, I, V, J, K, L, M, S were hypothyroid cases. The D, E, F, G, I, J, L, N, M, S cases were heterozygote (TC) and they had 3 bands: 162, 100 and 62 bp. While, A, B, C, H, K, O, P, Q, R cases were homozygote (TT) and has a single 162 bp band and the V case was homozygote (CC) with 100 and 62 bp bands and L band represented the Ladder.

### RFLP-PCR analysis of TSHR rs2268458 intronic polymorphism

The RFLP-PCR analysis of DNAs, extracted from hypo- and hyper-thyroid patients from Yazd province was carried out and as shown in Table 1, three types of genotypes were observed in present cases; homozygote (TT), heterozygote (TC) and homozygote (CC). Homozygote (TT) cases exhibit no disease symptoms, heterozygote (TC) cases show mild symptoms; while homozygote (CC) cases show severe thyroid dysfunction symptoms. According to the RFLP-PCR results, 59 cases were homozygotes (TT), 40 cases heterozygotes (TC) and 1 case was recorded to be homozygote (CC). In this study, there were 80 (*n* = 60 hypothyroid and *n* = 20 hyperthyroid) patient cases and 20 normal cases. A total of 25 cases comprised of homozygote (TT) hypothyroid females, 20 heterozygote (TC) hypothyroid females, and 7 homozygote (TT) hypothyroid females. While, 7 heterozygote (TC) hypothyroid males and 1 homozygote (CC) hypothyroid male case were also observed in this study. While, RFLP-PCR results showed 7 heterozygote (TT) hyperthyroid females, 8 cases of heterozygote (TC) hyperthyroid females, 3 homozygote (TT) hyperthyroid males and 2 heterozygote (TC) hyperthyroid males.

**Table 1:** Genotype analysis of hypo-and hyper thyroid male and female patients.

Number	Gender	Disease	Genotype	Number	Gender	Disease	Genotype	Number	Gender	Disease	Genotype
1	♀	Hypothyroid	TT	36	♂	Hypothyroid	TC	71	♀	Hyperthyroid	TC
2	♂	Normal	TT	37	♀	Hyperthyroid	TT	72	♂	Normal	TT
3	♀	Hypothyroid	TC	38	♀	Hypothyroid	TT	73	♀	Hypothyroid	TT
4	♀	Hypothyroid	TC	39	♀	Hypothyroid	TT	74	♀	Hypothyroid	TC
5	♂	Normal	TT	40	♂	Normal	TC	75	♀	Hypothyroid	TT
6	♀	Hypothyroid	TC	41	♂	Normal	TC	76	♂	Hypothyroid	TC
7	♀	Hypothyroid	TT	42	♀	Hypothyroid	TC	77	♂	Hypothyroid	TC
8	♀	Hypothyroid	TT	43	♂	Hypothyroid	TT	78	♀	Hypothyroid	TT
9	♀	Hypothyroid	TT	44	♀	Hypothyroid	TC	79	♀	Hypothyroid	TC
10	♀	Hypothyroid	TC	45	♀	Hypothyroid	TT	80	♀	Hypothyroid	TC
11	♂	Hyperthyroid	TT	46	♀	Hypothyroid	TT	81	♂	Hypothyroid	TT
12	♀	Hyperthyroid	TT	47	♀	Hypothyroid	TC	82	♀	Normal	TT
13	♀	Hypothyroid	TT	48	♀	Hypothyroid	TT	83	♀	Normal	TT
14	♀	Hypothyroid	TC	49	♂	Hypothyroid	TT	84	♀	Normal	TT
15	♀	Hyperthyroid	TT	50	♀	Hypothyroid	TC	85	♂	Hyperthyroid	TT
16	♀	Hyperthyroid	TC	51	♂	Hypothyroid	TT	86	♂	Hyperthyroid	TC
17	♀	Hypothyroid	TT	52	♀	Normal	TT	87	♂	Hyperthyroid	TT
18	♀	Hypothyroid	TT	53	♀	Normal	TT	88	♀	Normal	TT
19	♀	Hypothyroid	TT	54	♀	Normal	TT	89	♀	Hyperthyroid	TT
20	♀	Hypothyroid	TC	55	♀	Hypothyroid	TT	90	♀	Hyperthyroid	TC
21	♀	Hypothyroid	TT	56	♂	Hypothyroid	TC	91	♀	Normal	TT
22	♀	Hypothyroid	TT	57	♀	Hypothyroid	TC	92	♀	Hyperthyroid	TC
23	♀	Hypothyroid	TT	58	♀	Hypothyroid	TC	93	♀	Hyperthyroid	TT
24	♀	Hyperthyroid	TC	59	♂	Hyperthyroid	TC	94	♀	Normal	TT
25	♀	Hyperthyroid	TC	60	♀	Hypothyroid	TC	95	♀	Hyperthyroid	TC
26	♀	Hyperthyroid	TT	61	♀	Hypothyroid	TT	96	♀	Normal	TT
27	♀	Hypothyroid	TT	62	♀	Hypothyroid	TC	97	♀	Normal	TT
28	♀	Hypothyroid	TC	63	♂	Hypothyroid	CC	98	♀	Normal	TT
29	♀	Hypothyroid	TT	64	♀	Hypothyroid	TC	99	♀	Hypothyroid	TT
30	♂	Hypothyroid	TT	65	♂	Hypothyroid	TT	100	♀	Normal	TT
31	♀	Hyperthyroid	TC	66	♀	Hypothyroid	TC				
32	♀	Hypothyroid	TC	67	♂	Hypothyroid	TC				
33	♂	Hypothyroid	TT	68	♀	Normal	TC				
34	♀	Hypothyroid	TT	69	♀	Normal	TT				
35	♀	Hypothyroid	TC	70	♀	Hyperthyroid	TT				

**Figure 3:**
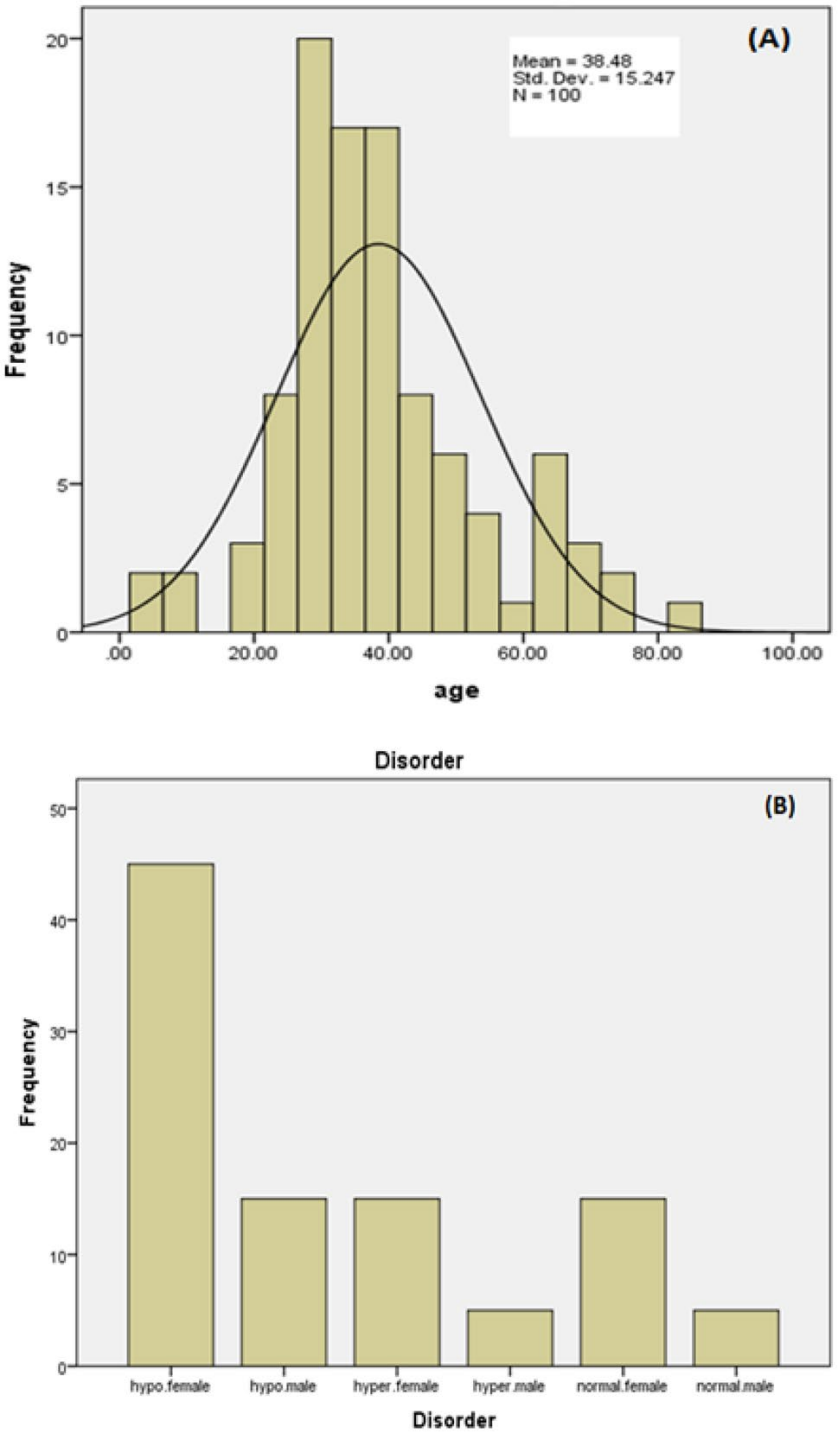
(**A**) The Histogram shows a transparent association between age and the risk of suffering from thyroid disorders. (**B**) Thyroid disorder frequency among male and female populations. Besides, hypothyroidism is more frequent than hyperthyroidism in all populations.

Besides, according to Table 1 and Fig. 3A,B, the analysis of hypothyroidism and hyperthyroidism was carried out in male and female populations, representing that the females were more likely to suffer from thyroid disorders. Moreover, hypothyroidism was predominant among study patients as compared to hyperthyroidism (*p* < 0.05).

Table 2 shows the association of individual's age and prevalence/severity of thyroid disorders. According to our results, older study population had had higher rate of thyroid disorders (*p* < 0.05). While, according to Table 3, the BMI of the individual was also directly related to the thyroid dysfunction. Our results suggest that hypothyroid patients were over-weight with BMI of 25–29.9 or obese with BMI of 30 or above. While, most of the hyperthyroid patients were underweight with BMI of < 18.5.

**Table 2:** Analysis of association of individual's age and thyroid disorders.

Number	Gender	Disease	Genotype	Age	Number	Gender	Disease	Genotype	Age	Number	Gender	Disease	Genotype	Age
1	♀	HYPO T	TT	38	36	♂	HYPO T	TC	42	71	♀	HYPER T	TC	38
2	♂	Normal	TT	27	37	♀	HYPER T	TT	30	72	♂	Normal	TT	41
3	♀	HYPO T	TC	32	38	♀	HYPO T	TT	35	73	♀	HYPO T	TT	30
4	♀	HYPO T	TC	70	39	♀	HYPO T	TT	73	74	♀	HYPO T	TC	30
5	♂	Normal	TT	35	40	♂	Normal	TC	36	75	♀	HYPO T	TT	84
6	♀	HYPO T	TC	34	41	♂	Normal	TC	64	76	♂	HYPO T	TC	62
7	♀	HYPO T	TT	54	42	♀	HYPO T	TC	22	77	♂	HYPO T	TC	59
8	♀	HYPO T	TT	29	43	♂	HYPO T	TT	30	78	♀	HYPO T	TT	74
9	♀	HYPO T	TT	31	44	♀	HYPO T	TC	28	79	♀	HYPO T	TC	24
10	♀	HYPO T	TC	34	45	♀	HYPO T	TT	31	80	♀	HYPO T	TC	25
11	♂	HYPER T	TT	40	46	♀	HYPO T	TT	40	81	♂	HYPO T	TT	8
12	♀	HYPER T	TT	43	47	♀	HYPO T	TC	37	82	♀	Normal	TT	33
13	♀	HYPO T	TT	49	48	♀	HYPO T	TT	43	83	♀	Normal	TT	62
14	♀	HYPO T	TC	28	49	♂	HYPO T	TT	40	84	♀	Normal	TT	37
15	♀	HYPER T	TT	41	50	♀	HYPO T	TC	27	85	♂	HYPER T	TT	42
16	♀	HYPER T	TC	38	51	♂	HYPO T	TT	5	86	♂	HYPER T	TC	47
17	♀	HYPO T	TT	65	52	♀	Normal	TT	40	87	♂	HYPER T	TT	51
18	♀	HYPO T	TT	71	53	♀	Normal	TT	56	88	♀	Normal	TT	25
19	♀	HYPO T	TT	25	54	♀	Normal	TT	56	89	♀	HYPER T	TT	34
20	♀	HYPO T	TC	7	55	♀	HYPO T	TT	37	90	♀	HYPER T	TC	39
21	♀	HYPO T	TT	33	56	♂	HYPO T	TC	42	91	♀	Normal	TT	24
22	♀	HYPO T	TT	30	57	♀	HYPO T	TC	27	92	♀	HYPER T	TC	31
23	♀	HYPO T	TT	23	58	♀	HYPO T	TC	37	93	♀	HYPER T	TT	43
24	♀	HYPER T	TC	32	59	♂	HYPER T	TC	48	94	♀	Normal	TT	17
25	♀	HYPER T	TC	19	60	♀	HYPO T	TC	41	95	♀	HYPER T	TC	29
26	♀	HYPER T	TT	36	61	♀	HYPO T	TT	50	96	♀	Normal	TT	23
27	♀	HYPO T	TT	34	62	♀	HYPO T	TC	33	97	♀	Normal	TT	35
28	♀	HYPO T	TC	69	63	♂	HYPO T	CC	54	98	♀	Normal	TT	31
29	♀	HYPO T	TT	46	64	♀	HYPO T	TC	45	99	♀	HYPO T	TT	35
30	♂	HYPO T	TT	41	65	♂	HYPO T	TT	20	100	♀	Normal	TT	30
31	♀	HYPER T	TC	40	66	♀	HYPO T	TC	35					
32	♀	HYPO T	TC	31	67	♂	HYPO T	TC	4					
33	♂	HYPO T	TT	30	68	♀	Normal	TC	66					
34	♀	HYPO T	TT	66	69	♀	Normal	TT	30					
35	♀	HYPO T	TC	33	70	♀	HYPER T	TT	47

**Table 3:** Analysis of association between patients’ BMI and thyroid disorders.

Number	Gender	Disease	Genotype	BMI	Number	Gender	Disease	Genotype	BMI	Number	Gender	Disease	Genotype	BMI
1	♀	HYPO T	TT	26	36	♂	HYPO T	TC	30.6	71	♀	HYPER T	TC	18
2	♂	Normal	TT	24.3	37	♀	HYPER T	TT	18.5	72	♂	Normal	TT	222
3	♀	HYPO T	TC	26.1	38	♀	HYPO T	TT	25.2	73	♀	HYPO T	TT	24
4	♀	HYPO T	TC	28	39	♀	HYPO T	TT	24.7	74	♀	HYPO T	TC	26.1
5	♂	Normal	TT	19.8	40	♂	Normal	TC	23.4	75	♀	HYPO T	TT	26.5
6	♀	HYPO T	TC	29	41	♂	Normal	TC	24	76	♂	HYPO T	TC	28.4
7	♀	HYPO T	TT	27.4	42	♀	HYPO T	TC	25.3	77	♂	HYPO T	TC	27.2
8	♀	HYPO T	TT	24.5	43	♂	HYPO T	TT	28.8	78	♀	HYPO T	TT	24.7
9	♀	HYPO T	TT	26.8	44	♀	HYPO T	TC	26.1	79	♀	HYPO T	TC	25.6
10	♀	HYPO T	TC	30.5	45	♀	HYPO T	TT	25.9	80	♀	HYPO T	TC	23.8
11	♂	HYPER T	TT	20	46	♀	HYPO T	TT	24.4	81	♂	HYPO T	TT	20.5
12	♀	HYPER T	TT	19	47	♀	HYPO T	TC	31.3	82	♀	Normal	TT	23.1
13	♀	HYPO T	TT	28.6	48	♀	HYPO T	TT	25	83	♀	Normal	TT	22.7
14	♀	HYPO T	TC	31	49	♂	HYPO T	TT	24.5	84	♀	Normal	TT	23.5
15	♀	HYPER T	TT	18.1	50	♀	HYPO T	TC	26	85	♂	HYPER T	TT	18.4
16	♀	HYPER T	TC	21.3	51	♂	HYPO T	TT	19	86	♂	HYPER T	TC	19.8
17	♀	HYPO T	TT	25	52	♀	Normal	TT	22.4	87	♂	HYPER T	TT	20.1
18	♀	HYPO T	TT	27.1	53	♀	Normal	TT	23.9	88	♀	Normal	TT	22.9
19	♀	HYPO T	TT	28.2	54	♀	Normal	TT	24.5	89	♀	HYPER T	TT	18.3
20	♀	HYPO T	TC	22	55	♀	HYPO T	TT	23.9	90	♀	HYPER T	TC	17.5
21	♀	HYPO T	TT	24.6	56	♂	HYPO T	TC	25.6	91	♀	Normal	TT	21.2
22	♀	HYPO T	TT	22.9	57	♀	HYPO T	TC	27.1	92	♀	HYPER T	TC	18.6
23	♀	HYPO T	TT	24.7	58	♀	HYPO T	TC	24.8	93	♀	HYPER T	TT	19
24	♀	HYPER T	TC	19	59	♂	HYPER T	TC	18.3	94	♀	Normal	TT	23.4
25	♀	HYPER T	TC	18.2	60	♀	HYPO T	TC	23.9	95	♀	HYPER T	TC	17.8
26	♀	HYPER T	TT	18.9	61	♀	HYPO T	TT	26.7	96	♀	Normal	TT	24.3
27	♀	HYPO T	TT	23	62	♀	HYPO T	TC	28.2	97	♀	Normal	TT	20.9
28	♀	HYPO T	TC	31.4	63	♂	HYPO T	CC	28.6	98	♀	Normal	TT	24
29	♀	HYPO T	TT	24.5	64	♀	HYPO T	TC	28	99	♀	HYPO T	TT	25.2
30	♂	HYPO T	TT	25.6	65	♂	HYPO T	TT	25.3	100	♀	Normal	TT	24.8
31	♀	HYPER T	TC	19	66	♀	HYPO T	TC	26.9					
32	♀	HYPO T	TC	26.3	67	♂	HYPO T	TC	19.5					
33	♂	HYPO T	TT	27.8	68	♀	Normal	TC	21.7					
34	♀	HYPO T	TT	27.4	69	♀	Normal	TT	22.6					
35	♀	HYPO T	TC	32.5	70	♀	HYPER T	TT	18.1					

### Allele and genotype analysis

**Figure 4:**
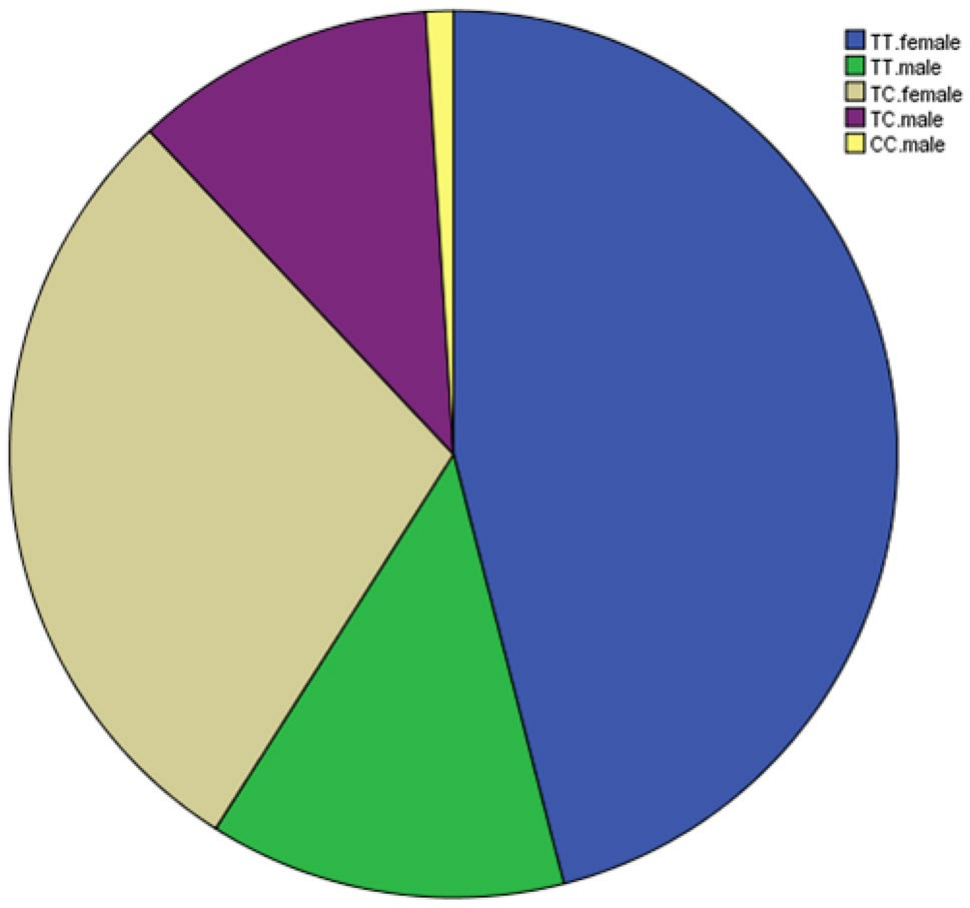
Prevalence of specific genotypes in male and female populations. Females with TT are > females with TC > males with TT ≥ males with TC > males with CC (And there are no females with CC).

The distribution of genotype frequencies for SNP s2268458 as case–control association analysis and all has been shown in Table 4 and Fig. 4. The major allele TT genotype in s2268458 of TSHR gene was observed in 42 patients and 17 normal individuals. While, the major allele CC genotype in s2268458 was observed in only 1 patient and the normal individuals did not show this genotype. Among 100 cases, 80 cases were patients and 20 cases were normal. In total, there were 59 TT, 40 TC and 1 CC. Among TT cases, 42 of them were patients and 17 of them were normal cases. Among TC cases, 37 of them were patients and 3 of them were normal cases. There was just one CC hypothyroid male case detected in our study. According to Table 5, Among the 100 tested cases, 75 cases were female and 25 cases were male. 59 cases were TT, 40 cases were TC and one case was CC. Among TT cases, 46 cases were female and 13 cases were male. Among TC cases, 29 cases were female and 11 cases were male. Just one case was CC that was a male. According to Table 6, our analysis on 60 hypothyroid and 20 hyperthyroid cases showed that 42 cases had TT genotype, 37 cases were TC and 1 case was CC. Among TT cases, 32 cases were hypothyroid and 10 cases were hyperthyroid. Among TC cases, 27 cases were hypothyroid and 10 cases were hyperthyroid. And one CC was observed to be hypothyroid.

**Table 4:** Observation of a specific genotype in normal and thyroid disorder cases.

	Genotype		
	TT	TC	CC
Patient	42	37	1
Normal	17	3	0
Total	59	40	1

**Table 5:** Observation of genotype according to patients’ gender.

	Genotype		
	TT	TC	CC
Female	46	29	0
Male	13	11	1
Total	59	40	1

**Table 6:** Observation of genotype according to hypothyroidism/hyperthyroidism disorder.

	Genotype		
	TT	TC	CC
Hypothyroid	32	27	1
Hyperthyroid	10	10	0
Total	42	37	1

## Discussion

During the present study, hypothyroidism was the most frequent thyroid problem among our patients but these results are not in accordance with all studies^22,23^ but has a coordination with some^24^. In Iran, legislation of using iodized salt was established in 1994, however goiter and urinary iodine concentration remained elevated in many Iranian provinces. A previous population-based study assessed the prevalence of hypothyroidism in Isfahan, Iran. They found that hypothyroidism was common (12.8% of women and 4.7% of men) and probably due to autoimmunity with no correlation to iodine intake^25^. However, whether this difference is due to geographic distribution of thyroid problems or genetic or environmental difference among nations should be elucidated in well-structured ecologic studies. Present study confirmed previous reports in which female gender was described as the most frequent one among hypothyroid and hyperthyroid patients. In our study, the mean age of women with MG was lower than men^26,27^, however, these results were not statistically significant. Our study did not show gender difference in ages less than 20, however, till 60 years of age, female gender was the most frequent one among patients and thereafter, men were the most common presenting gender too. These findings are in accordance with previous studies^19,28–30^. Since, 20–50 years is the reproductive period of women and before climacteric period, we hypothesize that the specific hormonal balance may have a role in presentation of clinical features of MG patients possibly as an exacerbating factor. Our study did not show any clinical presentation difference among MG patients with and without thyroid problem as well no para-clinical assessment EMG (electromyographic), RNS (Repetitive Nerve Stimulation), AchR-Ab (Acetylcholine Receptor Antibody)) difference was found between these two groups. Whether these findings are pure clinical finding or a consequent of not having enough study subjects remain to be elucidated by future studies with more patients. AITD is characterized by immunogenicity of the major thyroid antigens (Tg, TSHR and TPO)^29^. The study of intronic polymorphisms has been entertained because we now know that intronic DNA may be responsible for regulatory small RNAs as well as providing and/or influencing different start sites for TSHR mRNA generation^31,32^. The thyroid cells express a variety of TSHR mRNA splice variants, indicating that SNPs or small RNAs in this intronic DNA may be important in the generation of different receptor forms and/or their control. Recently, a study from Singapore demonstrated an association of a *TSHR* intron 1 SNP with GD. SNPs in intron 7 of the *TSHR* were also found to be associated with GD in Japanese, and SNPs in intron 1 of the *TSHR* were reported to be associated with GD in Caucasians^16^. So the result is, there is an association between *rs2268458* with hypothyroidism and it has been confirmed that the allele C has a pathogenic effect on recessive, dominant and co-dominant but the biggest effect is on recessive allele and it has been perceived that the allele C is a pathogenic allele, which is usually a recessive hereditary pattern^15^. In this study, homozygote cases (TT) will not show the symptoms and they only have a 162 bp band, heterozygote cases (TC) show some of the symptoms or some light symptoms and they have 3 bands: 162, 100 and 62 but the homozygote cases (CC), show sever symptoms and they have 2 bands 100 and 62 bp which agrees with the Iraq’s study who reported that TSHR rs2268458 polymorphisms were associated with hypothyroidism^15^ and disagrees with the Lebanon’s study who reported that TSHR rs2268458 polymorphisms were not associated with hypothyroidism and hyperthyroidism^26^.

According to^20^, there was a correlation between relapse of Graves' disease and CC genotype of TSHR gene on the rs2268458 of intron 1^20^. In a study, related to thyroid disease association with polymorphism in Iraq, 73 cases were homozygote (TT), 20 cases were heterozygotes (TC) and 1 case was homozygote (CC) and since all the research was done on females, the homozygote (CC) case was a woman^13^ but in this study, both men and females were involved and the homozygote case (CC) was a man. This shows a homozygote case (CC) can be seen in both females and men provided, both genders are studied and a large number of people are involved in the study.

## Conclusion

To our knowledge, this is the very first study showing an association of TSHR gene rs2268458 polymorphism with hypothyroidism and hyperthyroidism in Iran and particularly in Yazd province. In this study, it has been shown that *rs2268458* leads to a high risk in having hypothyroidism and hyperthyroidism, particularly hypothyroidism and since in yazd province some other factors such as smoking, stress, bad eating habits, not exercising and so on are high, *rs2268458* is more highlighted and it leads to more number of thyroid related diseases such as hypothyroidism and hyperthyroidism. Besides, it has been confirmed that thyroid disorders are more common among females as men.
